# Heart failure–concepts and significance. Birth of a prognostic model

**Published:** 2010-11-25

**Authors:** C Sinescu, L Axente

**Affiliations:** Department of Cardiology, ‘Bagdasar–Arseni’ Emergency Hospital, BucharestRomania

**Keywords:** heart failure, definition, descriptive terms, epidemiology, prognosis, mortality, hazard, survival function

## Abstract

Heart failure (HF) is a syndrome characterized by high prevalence in society, frequent hospitalization, reduced quality of life and high mortality (overall,50% of patients are dead at an interval of 4 years [1], annual mortality varying from 5% to 75%). Outcomes in heart failure are highly variable, prognosis of individual patients differs considerably and trial data, though valuable, does not often give an adequate direction. Taking into account the high prevalence of heart failure in society and its complexity physicians need a model to predict the risk of death, to estimate the survival of heart failure patients. A key element of interest in this area is the survival function, usually noted by S and defined as S(t)=exp(–H_0_(t)e^a^T^x^)=e^–H_0_(t)e^a^T^x^^

## Heart failure–Definition

Heart failure is a syndrome in which structural or functional cardiac conditions impair heart's ability to supply sufficient **blood flow** in order to meet the body's needs, or to do that at an elevated diastolic pressure[2].

There are many definitions of this complex syndrome, but none is satisfactory, due to the lack of a universally agreed definition and challenges in definitive diagnosis. Until now, only some selective features of this extremely complex physiological state were highlighted in the definitions–oxygen consumption, cardiac preload and afterload, left ventricular remodeling and dysfunction, ventricular filling pressures, neurohormonal responses, exercise capacity, etc. 

The new American and European guidelines and recommendations include new information and have the declared intention to simplify and clarify the previous recommendations[1].

Heart failure is a clinical syndrome in which patients have featured symptoms typical of heart failure (breathlessness at rest or on exercise, fatigue, tiredness, ankle swelling) and typical signs of heart failure (tachycardia, tachypnoea, pulmonary rales, pleural effusion, raised jugular venous pressure, peripheral oedema, hepatomegaly) and objective evidence of a structural or functional abnormality of the heart at rest (cardiomegaly, third heart sound, cardiac murmurs,abnormality on the echocardiogram, raised natriuretic peptide concentration)[1]. 

A clinical response to a pharmacological therapy directed to heart failure is not sufficient for the diagnosis of heart failure, although the usefulness/efficacy of the treatment may be established by the improvement in symptoms or signs (e.g. diuretic administration)[3]. 

Heart failure may be classified by structural abnormality (ACC/AHA), or by symptoms relating to functional capacity (NYHA).

ACC/AHA stages of heart failure (based on structure and damage to heart muscle)[4]

- Stage A: At high risk for developing heart failure. No identified structural or functional abnormality; no signs or symptoms.
- Stage B: Developed structural heart disease that is strongly associated with the development of heart failure, but without signs or symptoms.
- Stage C: Symptomatic heart failure associated with underlying structural heart disease.
- Stage D: Advanced structural heart disease and marked symptoms of heart failure at rest despite maximal medical therapy.

NYHA functional classification (severity based on symptoms and physical activity)[5] (NYHA classification refers to stages C and D)

- Class Ⅰ:No limitation of physical activity. Ordinary physical activity does not cause fatigue, palpitation, or dyspnoea.
- Class Ⅱ: Slight limitation of physical activity. Comfortable at rest, but ordinary physical activity results in fatigue, palpitation, or dyspnoea.
- Class Ⅲ: Marked limitation of physical activity. Comfortable at rest, but less than ordinary activity results in fatigue, palpitation, or dyspnoea.
- Class Ⅳ: Unable to carry on any physical activity without discomfort. Symptoms at rest. If any physical activity is undertaken, discomfort is increased.

## Descriptive terms in heart failure

### Acute and chronic heart failure

Acute heart failure (ICA) is a clinical syndrome caused by the action of a factor with brutal effect, often reversible, over the functional capacity of the heart.

Acute heart failure is defined by the rapid onset of signs and symptoms (secondary to cardiac dysfunction) resulting from impaired heart. It may occur in the presence or absence of preexisting heart disease. Acute heart failure may be an expression of systolic or diastolic dysfunction, heart rhythm abnormalities, or disturbances of preload or afterload. It is often a threat, life threatening, requiring emergency treatment.

Acute heart failure may present as acute de novo heart failure (a patient without known preexisting heart disease) or acute decompensation of chronic heart failure. In practice, the most common form is decompensation of chronic heart failure.

Classification of heart failure[1]

- New onset: First presentation, Acute or slow onset
- Transient: Recurrent or episodic
- Chronic: Persistent and Stable, worsening, or decompensated

Other forms of acute heart failure (ICA) include: acute heart failure with hypertension, pulmonary edema, cardiogenic shock, heart failure with increased cardiac output and right heart failure.

There is a number of well-known classifications that are used in the context of acute heart failure secondary to a myocardial infarction: Killip classification (designed to clinically assess the severity of myocardial dysfunction)[6] and Forrester classification (evaluate clinical and hemodynamic patients with acute myocardial infarction)[7]. In the original publication [8] therapeutic strategy depended on clinical and hemodynamic status – Forrester classification. Patients are classified according to signs of peripheral hypoperfusion (weak pulse, moist skin, cold, peripheral cyanosis, hypotension, tachycardia, confusion, oliguria) and to signs of pulmonary congestion (rales, chest X–ray changes). The mortality rate is different according to the class, respectively 2.2% in Class Ⅰ, 10.1% in Class Ⅱ, 22.4% Class Ⅲ, to 55.5% in  Class Ⅳ.

Killip classification [6]: Designed to provide a clinical estimate of the severity of circulatory derangement in the treatment of acute myocardial infarction.

- Stage Ⅰ: No heart failure. No clinical signs of cardiac decompensation (PCWP–estimate of left atrial pressure)
- Stage Ⅱ: Heart failure. Diagnostic criteria include rales, S3 gallop, and pulmonary venous hypertension. Pulmonary congestion with wet rales in the lower half of the lung fields.
- Stage Ⅲ: Severe heart failure. Frank pulmonary oedema with rales throughout the lung fields
- Stage Ⅳ: Cardiogenic shock. Signs include hypotension (SBP b90 mmHg), and evidence of peripheral vasoconstriction such as oliguria, cyanosis and sweating

Forrester classification[7]; Designed to describe clinical and haemodynamic status in acute myocardial infarction.

1. Normal perfusion and pulmonary wedge pressure
2. Poor perfusion and low PCWP (hypovolaemic)
3. Nearly normal perfusion and high PCWP (pulmonary oedema)
4. Poor perfusion and high PCWP (cardiogenic shock)

Another classification of severity of acute heart failure, used in some intensive care and coronary units, has been validated for cardiomyopathy[9],based on clinical signs[10], applicable in case of chronic decompensated heart failure[9].

Patients are divided into 4 classes based on two clinical criteria: assessment of perfusion/peripheral circulation and skin appearance and pulmonary congestion (appreciated by auscultation).

- Class Ⅰ warm skin without rales
- Class Ⅱ warm skin, rales present
- Class Ⅲ cold skin, without rales
- Class Ⅳ cold skin, rales present

This classification allows an accurate assessment of prognosis regarding the patients with cardiomyopathy[11].

Heart failure is a clinical and functional diagnosis, expression of supply / demand balance alteration; a relation in which both terms are equally important. A simple, objective, definition of chronic heart failure is difficult as long as there are no precise boundaries between ventricular and heart dysfunction (intracavitary pressure limit values, changes of flow, size and volume cavities)[3].

### Diastolic versus systolic heart failure 

The cardiologist's interest all over the world has been directed, for a long time, to the study of the systolic function of heart, being considered the key in the pathophysiology of heart failure. In the last decade, numerous studies have attempted to shed light on natural history, pathophysiology, diagnosis, prognosis and treatment of heart failure with diastolic dysfunction.

Epidemiological and clinical studies applied on hospital populations revealed that a percentage of 30–50% of patients with heart failure have preserved left ventricular function. Epidemiological data in patients with diastolic dysfunction are still very limited compared to available data on epidemiology of heart failure with systolic dysfunction, much more studied and well–documented.

Diastolic heart failure is characterized by 3 basic elements: the presence of signs and symptoms of heart failure, preserved left ventricular systolic function (ejection fraction >45%) and the presence of abnormal diastolic function (alterations of relaxation and / or compliance) [12].

We must make a clear delimitation between two different concepts: diastolic dysfunction and diastolic heart failure. Diastolic dysfunction characterized by abnormal mechanical properties and diastolic heart failure, is a clinical syndrome characterized by signs and symptoms of heart failure and evidence of altered diastolic function.

The concept of diastolic dysfunction defined by the existence of an abnormality of one or both of the constituent processes of diastole: relaxation and compliance. They may suffer elongation, delays or they may be incomplete, changes that can be measured with Doppler echography or cardiac catheterization[13]. These abnormalities of diastolic function may be detected in the presence or absence of the clinical syndrome of diastolic heart failure; they can also be noticed alone or with concomitant abnormalities of systolic function [14, 15].

Population studies estimate that approximately 5% of the general population have isolated diastolic dysfunction; systolic dysfunction has a similar rate, about half being asymptomatic[16].

The main etiology of heart failure in developed societies is ischemic heart disease. In this context, many signs of heart failure are associated with left ventricular systolic dysfunction, although diastolic impairment at rest is usually present[3].

Diastolic dysfunction in young patients is rare (about 15% in populations under 50 years), but it increases in importance in the elders (50% in patients over 70 years) [17–22].

Diastolic dysfunction is more common in women in whose systolic hypertension and myocardial hypertrophy contribute to cardiac dysfunction[23, 24].

Although most patients with acute pulmonary edema have a systolic dysfunction, many cases of developing a clinical picture of acute pulmonary edema in patients with diastolic heart failure with preserved systolic function are described in literature[25].

Among the patients with chronic heart failure, a significant percentage has diastolic heart failure with preserved systolic function, if we take into account left ventricular ejection fraction at rest[23, 26].

Diastolic and systolic heart failure cannot be considered as separate pathophysiological entities, they coexist in most patients with the dominance of one or the other[3]. Most patients with systolic dysfunction associate changes in diastolic function [3]. In some patients with hypertension or hypertrophic cardiomyopathy, the dominant opinion is that diastolic dysfunction and abnormalities of relaxation and compliance are rather an earlier and more sensitive marker of cardiac damage than the alteration of systolic function, measured by decreased ejection fraction[3, 23, 27].

### Other descriptive terms in heart failure

A number of other descriptive terms was frequently used in the past, but now they are used only occasionally, their clinical value being reduced. These terms do not provide information on the etiology and they have a reduced influence in establishing a modern heart failure therapy.

The terms of right and left heart failure, forward and backward HF, high and low output HF, mild, moderate, or severe HF are old terms used somehow arbitrary, expressing an imprecise measure of this complex clinical syndrome.

Currently, the assessment of dyspnea and the limitation of the daily work are done by classifying the patient in functional class NYHA–New York Heart Association and the decline of functional assessment is performed using the Katz ADL Scale-Activities of Daily Living[28].

## Descriptive epidemiology

### The importance and extent of the problem. Incidence. Prevalence

Heart failure is a clinical syndrome, which, epidemiologically, is in a continuous growth in the economic developed countries and the developing ones, in contrast to ischemic heart disease, which is declining in the developed countries, due to the prevention programs. This fact is due to an aging population and to significant advances in the diagnosis and pharmacological treatment of heart disease.

Congestive heart failure (CHF) is a major public healthcare problem, being an invalidating condition, with a bad long–term prognosis, which reduces the quality of life and has a considerable economic cost to the individual and the society in general.

Current estimates show that CHF affects over 5 million people in the U.S., approximately 2% of adults and 10% of elderly people beeing affected by this ‘cardiovascular epidemic’ which was designated as national research priority[29].

With an incidence of over 400,000 new cases diagnosed each year[17], and approximately 1 million hospital admissions annually, out of which over 80%  patients aged over 65 years[17, 30], heart failure is the only major cardiovascular disease that increases in the United States.

Today it is estimated that the prevalence in the European countries ranges between 0.4 and 2%[31], the studies estimating that about 14 million of the approximately 900 million inhabitants of the 51 European countries suffer from heart failure.

The prevalence of heart failure increases with age[32], the average age of this population being 74–75 years and it is going due to increase in time, the data for the elderly population show percentage increased prevalence of disease[33–36].

The large number of studies carried out on large lots of heart failure patients shows the attention given to heart failure in clinical practice. We must note that most studies that refer to therapeutic management addresses to patients with heart failure and systolic disfunction, fewer to patients with preserved systolic function and heart failure.

In the last decade special attention has been paid to diastolic heart failure[30],  pathophysiological condition in which heart failure syndrome is caused by abnormalities of diastolic ventricular function. Epidemiological and clinical studies conducted on hospital populations revealed that a percentage of 30–50% of patients with CHF have preserved left ventricular function. Epidemiological data on diastolic dysfunction are still very limited compared to available data on epidemiology of heart failure with systolic dysfunction, carefully studied and well–documented.

Diastolic heart failure receives increasing interest from clinicians around the world because its clinical and epidemiological significance proved to be growing[37]. In the last decade a series of data has been accumulated that  provide conclusive information on the epidemiology of diastolic dysfunction, about its magnitude and complexity. The studies performed in the recent years revealed an annual mortality rate of patients with preserved systolic function heart failure of 8–17%[38] and 9–28%[39]. Although this is a significantly lower mortality rate than that of patients with heart failure and systolic dysfunction (about half), it represents 3–4 times the mortality of patients with the same age enrolled in control group[39]. 

### Age and prevalence of heart failure 

As mentioned above the prevalence of heart failure increases with age[30], the average age of this population being 74–75 years. Because of the increasing proportion of elderly population an increase in the prevalence of this disease is registrated[33–36]. Data from literature show an increase in prevalence from 6.6% in 65–69 years age group to 14% in over 85 years age group[29].

Looking at the percentage of patients with diastolic heart failure from the  people with symptomatic CHF we can notice a clear increase with age. Diastolic HF percentage varies from a rate of approximately 15% in populations under 50 years, to 33% in the 50–70 years age group and 50% in patients over 70 years[17, 18, 21, 22, 40, 41].

Recent clinical studies in patients with CHF and population observations estimate that heart failure with preserved systolic function represents  approximately 30–50% of cases hospitalized for symptomatic heart failure and more than 50% for people over 65 years[26, 42–44].

Increasing prevalence of diastolic dysfunction in the elderly people is due to: age related to physiological changes (increased interstitial fibrosis and myocardial hypertrophy, which causes alteration of ventricular relaxation and compliance) and induced pathophysiological changes associated comorbidities – essential hypertension (most important factor), ischemic heart disease, diabetes and left ventricular hypertrophy. With age there is a higher prevalence of these factors which may explain an increased percentage of patients with distolic dysfunction in the elderly people[45]. The risk factors mentioned above determine structural and functional heart adaptations that along with age–induced changes lead to alterations in diastolic function.

### Sex, etiology and the prevalence of heart failure 

In younger age groups, patients under 50 years, heart failure as a whole (patients with systolic dysfunction and diastolic dysfunction) is more common in men. At this age the most common etiology is ischemic coronary disease, more common in males. The main causes of heart failure are: coronary heart disease (70% of patients with HF), valve disease and cardiomyopathies each with approximately 10% of cases. In elderly people prevalence is equal between the sexes[1].

Numerous epidemiological reports have noticed that women with heart failure have an increased percentage (than men) of preserved systolic function[40, 44, 46, 47].

In similar age groups and associated clinical conditions female patients have more diastolic heart failure than men. Out of the 2.4 million female patients with heart failure in the U.S. more than 50% of them have normal systolic function [40].

There is no registry of heart failure in Romania as there is in other countries, so there is no precise data on the epidemiology of heart failure. Older data estimate a heart failure prevalence of 0.2–0.5% in the general population, that means approximately 150,000–200,000 patients with heart failure diagnosis[48]. Recent data from Statistics by Country for congestive Heart Failure[49] estimate an incidence of 0.146% (32 875) cases of heart failure and a prevalence of 1.76% (394 509) cases of heart failure in Romania. The President of the Romanian Heart Failure Working Group estimates the number of heart failure patients, at approximately 800–900 000, a number that increases permanently[50].

## Morbidity. Quality of life. Costs

HF morbidity is particularly important, patients with heart failure require frequent medical visits at home or rehospitalization, which represent a significant expenditure of health resources. In the first year after hospital discharge approximately 50% of patients with heart failure require rehospitalization, the data are similar in those with systolic dysfunction or diastolic dysfunction.

Heart failure is the main cause of hospitalization in the Medicare population in the USA[51]. Data from Scotland show that the number of hospitalizations in which heart failure is primary or secondary diagnosis of hospitalization is increasing in Europe. According to a hospital record, 4.7% of women admissions and 5.1% of men admissions are due to heart failure (at any time to diagnose it)[52]. While only a few cases are due to acute heart failure, first presentation, most cases are due to decompensation of chronic heart failure. The HF incidence, regardless of the degree of severity, varies between 2.3 and 3.7 per 1000 inhabitants per year[53–54].

Epidemiological data show that morbidity is decisively influenced by age,  rehospitalization rate (at one year) increases from 25% for under 50 years age group, to 50% for 50–70 years age group [18]. 

Some European studies show that about 1% of the national health budget is allocated to the therapy of heart failure, in the U.S. about 2% of the national health budget is allocated to this problem. The large number and the long length of hospitalization for acute heart failure or decompensation of chronic heart failure are a substantial economic burden for the healthcare budgets. In U.S., the first cause of hospitalization in patients over 65 years is heart failure. In Scotland, the number of patients with heart failure in hospital increased by 60% between 1980 and 1990. The United States spends about  20 billion dollars annually, 10% of healthcare budget allocated for the management of cardiovascular disease with heart failure, 75% of the amount being allocated to hospital care. Heart failure is the most expensive cardiological syndrome [56,57].

## Prognosis. Mortality

Prognosis is heterogeneous and depends on the heart failure class, its etiology and the patient's age. The prognosis of untreated heart failure is unknown. Multiple studies and metaanalyses highlighted various predictive criteria: clinical–radiological (NYHA functional class and heart size on chest X–ray examination), presence of atrial fibrillation, ejection fraction of VS, maximal O2 consumption during exercise, 6–minute walk test, pulmonary capillary pressure, serum catecholamine, natriuretic peptides and ventricular arrhythmias.

The latest ESC guidelines summarize the knowledge about the conditions associated with a poor prognosis in heart failure:

**Table 1:** Conditions associated with a poor prognosis in heart failure–according to ESC guidelines 2008[1](*Powerful predictors)

Demographics	Clinical	Electrophysiological	Functional/exertional	Laboratory	Imaging
Advanced age*	Hypotension*	Tachycardia Q waves	Reduced work, low peak	Marked elevation of BNP/NT pro–BNP*	Low LVEF*
Ischaemic etiology*	NYHA functional class Ⅲ–Ⅳ*	Wide QRS*	VO2*	Hyponatraemia*	Increased LV volumes
Resuscitated sudden death*	Prior HF hospitalization*	LV hypertrophy	Poor 6–minute walk distance	Elevated troponin*	Low cardiac index
Poor compliance	Tachycardia	Complex ventricular arrhythmias*	High VE/VCO2 slope	Elevated biomarkers, neurohumoral activation*	High LV filling pressure
Renal dysfunction	Pulmonary rales	Low heart rate variability	Periodic breathing	Elevated creatinine/BUN	Restrictive mitral filling pattern, pulmonary hypertension
Diabetes	Aortic stenosis	Atrial fibrillation		Elevated bilirubin	Impaired right ventricular function
Anaemia	Low body mass index	T–wave alternations		Anaemia	
COPD	Sleep–related breathing disorders			Elevated uric acid	
Depression					

Framingham's study reported a 65% survival rate at 1 year after specifying the diagnosis of heart failure, respectively 25% in men, and, 35–40% in women, meaning 5 years survival.  Another study–Mayo Clinic–Minnesota, shows a similar 66% one–year survival after the diagnosis indication. The main causes of death are sudden death or terminal heart failure (50% of the cases). Depending on the functional NYHA class in which the patients are reported, different mortality rates are recorded: CONSENSUS Ⅰ studies elderly patients with heart failure; NYHA class ⅓, reports a mortality rate of 44% at 6 months, and the ⅤHeft and SOLVD study shows a mortality rate of 15–20% at 1 year, in patients in NYHA class Ⅱ and Ⅲ. 

Patients with acute heart failure have a very severe prognosis, large randomized trials with hospitalized patients for decompensated heart failure have shown a 9.6% mortality rate at 60 days and a 35.2% combined mortality rate and rehospitalization at 60 days[58, 59]. 

Ischemic etiology proves to be a negative prognostic factor in AIRE and TRACE studies on patients with post–myocardial infarction heart failure, a 14–25% mortality rate in the 1 year registered in these patients. Mortality is particularly high in patients with acute myocardial infarction associated with severe HF, with a mortality rate close to 30% in the 1 year[59, 60].

Important observational studies that include patients with both systolic and diastolic heart failure show that although short–term mortality rate may be lower in patients with diastolic dysfunction, long–term survival analysis shows a similar mortality rate for both groups of patients, especially those over 65 years[14, 46]. Different results regarding mortality in diastolic heart failure may be explained by differences in etiology and age of the patients taken into survey.

Another determinant of mortality is the age, mortality in diastolic heart failure increases significantly with age. The data show that mortality at 5 years is 15% in below the 50–year–old group, 33% in 50–70 year–old group, reaching 50% in patients over 70 years old. Thus, in the elderly people over 70 years old the mortality rate for heart failure diastolic and heart failure systolic is practically equivalent[15, 22, 46].

Patterns of morbidity and mortality in Romania have undergone significant changes in the recent decades, due to the increased incidence and prevalence of chronic disease and mortality associated with these causes, in the context of the increasing percentage of elderly population, coupled with the action of multiple biological, environmental, behavioral risk factors as well as the influence of the socio–economic conditions and healthcare.

Compared to the average of the first 15 states included in the European Union (EU–15) and the average of the new member states (NMS–10), the overall mortality, has shown a slow downward trend (EU–15 average was 9.93 deaths per 1000 inhabitants in 1999 and 9.67 deaths per 1000 inhabitants in 2001, and the NMS–10 average was 10.78 deaths per 1,000 inhabitants in 1999 and 10.36 deaths per 1,000 inhabitants in 2002)[61]. Romania is recording a substantial increase in overall mortality rate, which, combined with the dynamics of birth and fertility leads to a long–term aging population process, with major negative consequences for both the healthcare system and the social security one.  

There are not sufficient epidemiological data yet  on diastolic heart failure in Romania, some studies show a high mortality rate, at 1 year up to 29% in the diastolic heart failure lot and 33% in systolic (note patients included in the study are severe cases that required hospitalization in an emergency hospital)[62].

Prognosis of individual patients differs considerably, outcomes in highly variable trial data often do not give an adequate direction; taking into consideration the magnitude of this syndrome in the society and its complexity, we need a model to predict the risk of death, to estimate survival of heart failure patients. 

Survival analysis is a branch of statistics dealing with the life eextent of biological organisms. More generally, the survival analysis involves estimating the duration of survival until a certain event. In this context, in the specialized literature devoted to survival analysis, death is considered an ‘event’. Survival analysis seeks to answer the questions related to the survival of an individual or a lot from a population, over a certain period of time, and which are the causes of increase or decrease in the extent of survival.

A key element in this area is the **survival function**, usually noted by S and defined as: S(t)=Pr(T>t) in which–t represents one moment in time;	T is a random variable designating the time (or age) of death;Pr() notes the probability of occurrence of an event. Pr(T>t) means the probability that the time of death T occurs some time after the specified time, t.

Usually, survival function S(t) has the following two properties: S(0)=1; S(t_2_)< S(t_1_) for the moments of time t_2_>t_1_. Last property shows that the survival function is monotonously decreasing in time. Survival at a later age is only possible if the subject has survived to all younger ages. Also, for the usual mathematical functions used for survival models, the value of S (t) function tends to 0 (zero) at a time when t tends to infinity.

In connection with the survival function we can define the **lifetime distribution function** (life extension) by the relation: F(t)=Pr(T<t)=1–S(t). Pr(T<t) means the probability that death time T occurs before or at a specified time t, at the latest.

**Figure 1:**
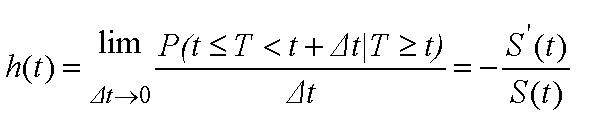
Hazard Function

Another extremely important concept is the **hazard function**, conventionally denoted by h (t). Hazard function can be defined in mathematical terms as in the relation Figure 1:

in which S'(t) represents time derivative of survival function. Hazard function is the mortality rate at time t conditioned by survival until time t or later (T>t). Hazard function is non negative, this meaning that h(t)>0.

**Figure 2:**
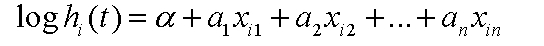
A parametric model of the hazard function logarithm, based on a multiple linear distribution

**Figure 3:**
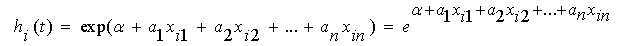
Another form of the hazard function logarithm, based on a multiple linear distribution

Survival models commonly use hazard function or hazard function logarithm. For example, a parametric model of the hazard function logarithm, based on a multiple linear distribution can be written as Figure 2 or Figure 3:

In these equations the index i represents the number of observation, the index n is the number of independent variables noted x_i1_, x_i2_, …, x_in_ and the a_1_, a_2_,…, a_n_, are the coefficients of the model. In this relation alpha is a kind of reference function/value (baseline function), because logh_i_(t)=alpha when all independent variables are zero. The purpose of multiple regression is to highlight the relationship between a dependent variable and many independent variables (control variables, predictors).

One of the most used functions of survival is the Cox multiple regression model [2], proportional hazards model.

**Figure 4:**
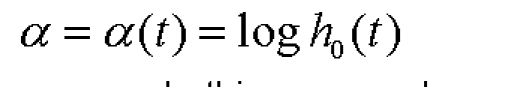
Cox's survival model

In this case we have:

**Figure 5:**
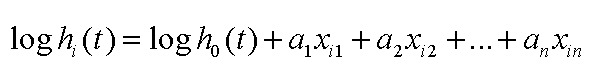


**Figure 6:**
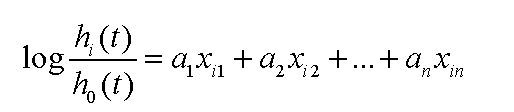


which will result in a hazard functionFigure 8:

**Figure 7:**
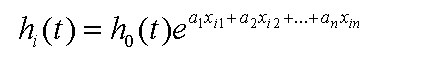


h_0_(t)is called baseline hazard function. In Cox's model it is unspecified.

Taking two observations, i and j in equationFigure 8, the following formula results

**Figure 8:**
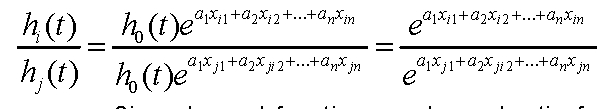


Since hazard functions as hazard ratio for any pair of observations, according to Figure 8, it does not depend on time t, Cox's model is often referred to as proportional hazards model. 

**Figure 9:**
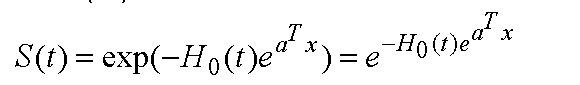


Cox's model can be actually extended to include time–dependent variable x. In this case, however, Cox's model is no longer a proportional hazards model. In this paper as in the vast majority of used applications, Cox's model is used for variable x independent of time. The equation Figure 8 can be written in the general form Figure 9:

in which a is n–dimensional column's vector of model parameters, with components a_1_, a_2_,…, a_n_, x is n–dimensional column's vector of independent variables with components x_1_,x_2_, …, x_n_, and T designates the sign of transposition of a vector.

**Figure 10:**
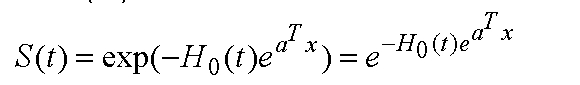
Cumulative hazard function

Survival function for Cox's proportional hazards model Figure 9 is Figure 10

**Figure 11:**
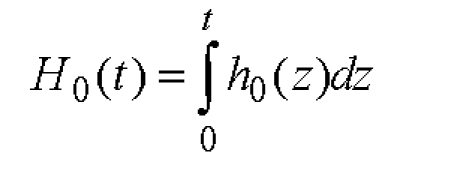


For baseline hazard function h_0_(t) respectively for H_0_(t) we can determine the values on experimental bases or we may use known statistical models: exponential, Weibull or Gompertz[64].

To determine the major factors that allow the forecasting survival (or mortality) we may use Cox's multiple regression model, the proportional hazards model[63].

The next step is to fit the requested regression model to all data and to create a heart failure prognostic model. 

The number of individuals living with heart failure is steadily increasing and there are many applications of such a model that may estimate the survival of a patient with heart failure.

## References

[1] Dickstein K (2008). Acute and Chronic Heart Failure (Diagnosis and Treatment), ESC Clinical Practice Guidelines. Eur Heart J.

[2] Zipes L, Braunwald B (2004). International edition.

[3] Swedberg K (2005). Guidelines for the diagnosis and treatment of chronic heart failure: full text. The Task Force for the Diagnosis and Treatment of Chronic Heart Failure of the European Society of Cardiology. European Heart Journal.

[4] Hunt SA (2005). American Heart Association. Circulation.

[5] Brown L (1994). The Criteria Committee of the New York Heart Association. Nomenclature and Criteria for Diagnosis of Diseases of the Heart and Great Vessels.

[6] Killip T, Kimball JT (1967). Treatment of myocardial infarction in a coronary care unit. A two year experience with 250 patients. Am J Cardiol.

[7] Forrester  JS, Diamond GA, Swan HJ (1977). Correlative classification of clinical and hemodynamic function after acute myocardial infarction. Am J Cardiol.

[8] Forrester (1977). Am J. Cardiol.

[9] Nohria A, Lewis E, Stevenson LW (2002). Medical management of advanced heart failure. JAMA.

[10] Grady KL, Kennedy  G, Moser DK (2000). AHA Scientific Statement: Team management of patients with heart failure: A Statement of health care professional from the cardiovascular nursing council of the American Heart Association. Circulation.

[11] Nohria A, Fang JC (2003). Clinical assessment identifies hemodynamic profiles that predict outcomes in patients admitted with heart failure. JACC.

[12] Working Group Report (1998). How to diagnose diastolic heart failure. Eur Heart J.

[13] Banerjee P, Banerjee T, Khand A (2002). Diastolic heart failure: neglected or misdiagnosed?. J Am Coll Cardiol.

[14] Smith GL, Masoudi FA, Vaccarino V (2003). Outcomes in heart failure patients with preserved ejection fraction. J Am Coll Cardiol.

[15] Zile MR (2003). Heart failure with preserved ejection fraction: is this diastolic heart failure? Editorial comment. J Am Coll Cardiol.

[16] Rodeheffer RJ (2002). Epidemiology and screening of asymptomatic left ventricular dysfunction. J Cardiac Fail.

[17] Dauterman KW, Massie BM, Gheorgiade M (1998). Heart failure associated wih preserved systolic function: A common and costly clinical entity. Am Heart J.

[18] Zile MR, Brutsaert DL (2002). New concepts in diastolic dysfunction and diastolic heart failure: Part 1. Diagnosis, prognosis, and measurements of diastolic function. Circulation.

[19] Senni M, Tribouilloy CM, Rodeheffer RJ (1998). Congestive heart failure in the community: a study of all incident cases in Olmsted County, Minnesota, in 1991. Circulation.

[20] Gottdiener JS, Arnold AM, Aurigemma GP (2000). Predictors of congestive heart failure in the elderly: the Cardiovascular Health Study. J Am Coll Cardiol.

[21] Aurigemma GP, Gottdiener JS (2001). Predictive value of systolic and diastolic function for incident congestive heart failure în the elderly: the Cardiovascular Health Study. J Am Coll Cardiol.

[22] O'Connor CM, Gattis WA, Shaw L (2000). Clinical characteristics and long–term outcomes of patients with heart failure and preserved systolic function. Am J Cardiol.

[23] Cleland JG, Swedberg K, Follath F (2003). The Euro– Heart Failure survey programme – a survey on the quality of care among patients with heart failure in Europe. Part 1:patient characteristics and diagnosis. Eur Heart J.

[24] McMurray J, Swedberg K (2004). Heart failure with preserved left ventricular systolic function. J Am Coll Cardiol.

[25] Gandhi SK, Powers JC (2001). The pathogenesis of acute pulmonary edema associated with hypertension. N Engl J Med.

[26] Vasan RS, Benjamin EJ, Levy D (1995). Prevalence, clinical features and prognosis of diastolic heart failure:an epidemiologic perspective. J Am Coll Cardiol.

[27] McDonagh TA, Morrison CE (1997). Symptomatic and asymptomatic left–ventricular systolic dysfunction in an urban population. Lancet.

[28] Katz S, Down TD, Cash HR (1970). Progress in the development of the index of ADL. Gerontologist.

[29] Kitzman DW (2002). Diastolic heart failure in the elderly. Heart Fail Rev.

[30] Pernenkil R, Vinson JM, Shah AS Course and prognosis in patients 70 years of age with congestive heart failure and normal versus abnormal left ventricular.

[31] Masterd A, Hoes AW, de Bruyne  MC (1999). Prevalence of heart failure and left ventricular dysfunction in the general population. Eur Heart J.

[32] McKee PA, Castelli WP, McNamara PM (1971). The natural history of congestive heart failure: the Framingham study. N Engl J Med.

[33] McMurray J, McDonagh T, Morrison CE (1993). Trends in hospitalization for heart failure in Scotland 1980–1990. Eur Heart J.

[34] Murdoch DR (1998). Importance of heart failure as a cause of death. Changing contribution to overall mortality and coronary heart disease mortality in Scotland 1979–1992. Eur Heart J.

[35] Cleland JG, Gemmell I (1999). Is the prognosis of heart failure improving?. Eur J Heart Fail.

[36] Cleland JG, Khand A, Clark A (2001). The heart failure epidemic:exactly how big is it?. Eur Heart J.

[37] Toporan D, Rovai D (2006). Semnificatia clinica si epidemiologica a insuficientei cardiace diastolice. Romanian Heart Journal.

[38] Sweitzer NK (2000). Diastolic heart failure: miles to go before we sleep. Am J Med.

[39] Vasan RS, Benjamin EJ (2001). Diastolic heart failure–no time to relax. N Engl J Med.

[40] Senni M, Tribouilloy CM, Rodeheffer RJ (1998). Congestive heart failure in the community: a study of all incident cases în Olmsted County, Minnesota, in 1991. Circulation.

[41] Gottdiener JS, Arnold AM, Aurigemma GP (2000). Predictors of congestive heart failure in the elderly: the Cardiovascular Health Study. J Am Coll Cardiol.

[42] Kitzman DW, Gardin JM (2001). Importance of heart failure with preserved systolic function in patients 65 years of age. Am J Cardiol.

[43] Cohen–Solal A (2002). Diastolic heart failure: myth or reality?. Eur J Heart Fail.

[44] Devereux RB, Roman MJ, Liu JE (2000). Congestive heart failure despite normal left ventricular systolic function in a population–based sample: The Strong Heart Study. Am J Cardiol.

[45] Fischer M (2003). Prevalence of left ventricular diastolic dysfunction in the community:Results from a Doppler echocardiographic–based survey of a population sample. Eur Heart J.

[46] Philbin EF (2000). Systolic versus diastolic heart failure in community practice:clinical features, outcomes, and the use of angiotensin–converting enzyme inhibitors. Am J Med.

[47] Richardson LG (2001). Women and heart failure. Heart and Lung.

[48] Comisia de Cardiologie (2000). Insuficienta cardiaca, ghid de diagnostic si tratament. Ghiduri de practica medicala.

[49] US Census Bureau LB (2004). Population Estimates, 2004 and US Census Bureau, International Data Base, 2004 Congestive Heart Failure, Statistics by Country for Congestive Heart Failure.

[50] Macarie C (2003). Congresul National de Cardiologie.

[51] Ghali JK (1990). Trends in hospitalization rates for heart failure in the United States, 1973–1986. Evidence for increasing population prevalence. Arch Intern Med.

[52] Stewart S (2001). Trends in hospitalization for heart failure in Scotland, 1990–1996. An epidemic that has reached its peak?. Eur Heart J.

[53] Remme WJ (2001). Guidelines for the diagnosis and treatment of chronic heart failure. Eur Heart J.

[54] Felker GM (2003). The problem of decompensated heart failure: nomenclature, classification, and risk stratification. Am Heart J.

[55] Cowie MR (1997). The epidemiology of heart failure. Eur Heart J.

[56] Adams KF (1998). Clinical definition and epidemiology of advanced heart failure. Am Heart J.

[57] O'Connell JB (1998). The economic burden of heart failure. Clin Cardiol.

[58] Cleland JG (2003). The Euroheart failure survey programme–a survey on the quality of care among patients with heart failure in Europe. Part 1: patient characteristics and diagnosis. Eur Heart J.

[59] Investigators A (1993). Effect of ramipril on mortality and morbidity of survivors of acute myocardial infarction with a clinical evidence of heart failure. Lancet.

[60] Stevenson R (1993). Short and long term prognosis of acute myocardial infarction since introduction of thrombolysis. BMJ.

[61] Publicatiile Institutului National de Statistica, Lucrarile Centrului National pentru Organizarea si Asigurarea Sistemului Informational si Informatic in Domeniul Sanatatii Bucuresti (2007). European region.

[62] Toporan D (2005). The less known face of heart failure in elderly: the prevalence and the prognosis implications of preserve versus impaired systolic function. The 5th Congress of the European Federation of Internal Medicine.

[63] Cox DR (1972). Regression Models and Life Tables (with Discussion). Journal of the Royal Statistical Society.

[64] Bender R (2005). Generating survival times to simulate Cox proportional hazards models. Statistics in Medicine.

